# Utilizing Artificial Intelligence for CSF Segmentation and Analysis in Head CT Imaging: A Systematic Review

**DOI:** 10.3390/brainsci15111144

**Published:** 2025-10-25

**Authors:** Michał Bielówka, Adam Mitręga, Dominika Kaczyńska, Marcin Rojek, Mikołaj Magiera, Jakub Kufel, Sławomir Grzegorczyn

**Affiliations:** 1 Students’ Scientific Association of Computer Analysis and Artificial Intelligence, Department of Radiology and Nuclear Medicine, Medical University of Silesia in Katowice, 40-752 Katowice, Poland; 2 Department of Biophysics, Faculty of Medical Sciences in Zabrze, Medical University of Silesia, 19 H. Jordan Str., 41-808 Zabrze, Poland; 3 Department of Radiology and Nuclear Medicine, Faculty of Medical Sciences in Katowice, Medical University of Silesia, Medyków 14, 40-752 Katowice, Poland

**Keywords:** cerebrospinal fluid, artificial intelligence, machine learning, image segmentation

## Abstract

**Background**: The intracranial space has limited capacity; thus, volume changes in any component can raise intracranial pressure and cause mass effect. This mechanism underlies many neurological disorders. Artificial Intelligence, increasingly applied in medicine and diagnostic imaging, may support the evaluation of such conditions. This systematic review investigates AI-based models for cerebrospinal fluid segmentation and analysis on computed tomography. **Methods**: In December 2024, a systematic review was conducted across MEDLINE (PubMed), Scopus, Web of Science, Embase, and Cochrane Library. From 559 identified studies, 14 were included after independent review by two evaluators. Extracted data covered study characteristics, AI model design, dataset composition, and performance metrics for CSF segmentation. Quality assessment followed PRISMA 2020 and used JBI, AMSTAR 2, and CASP checklists. **Results**: The 14 studies demonstrated applications of AI in CSF segmentation and volumetric assessment, primarily for hydrocephalus diagnosis, mass effect evaluation, and stroke outcome prediction. Convolutional Neural Networks and Random Forests were the most frequent approaches. Reported segmentation accuracy was high, with Dice Similarity Coefficient values ranging from 0.75 to 0.95 and strong volumetric correlations (r up to 0.99) between AI-based and manual measurements. **Conclusions**: AI-assisted CSF segmentation from CT images shows promising accuracy and efficiency, with potential to enhance neurological diagnostics. Remaining challenges include dataset variability, inconsistent algorithm performance, and limited clinical validation. Future research should prioritize standardization of methods, larger and more diverse training datasets, and integration of AI tools into clinical workflows.

## 1. Introduction

The intracranial space has limited capacity, which means that any changes in the volume of its components—such as brain tissue, blood, or cerebrospinal fluid (CSF)—can cause serious health consequences [1,2]. In many neurological conditions, including brain tumors, strokes, neuroinflammatory disorders and traumatic brain injuries, a reduction in CSF reserve and the development of mass effect may occur, potentially resulting in severe complications [3,4]. Consequently, the assessment of CSF volume and the monitoring of its distribution are becoming critical elements of neuroimaging diagnostics.

In recent years, Artificial Intelligence (AI) has played an increasingly significant role in the analysis and interpretation of medical data. Through advanced algorithms, AI can automate the analysis of medical images and the identification of abnormalities in CSF volume and distribution [5,6,7]. Deep neural networks (DNNs) such as U-Net and its variants are the most commonly employed machine learning techniques in the segmentation of medical images and the analysis of CSF distribution [8]. These architectures are capable of accurately extracting anatomical structures due to their convolutional layers and ‘skip connections’, which help preserve spatial information [9]. In addition to convolutional networks, classical machine learning algorithms are also used—such as the random forest method, which classifies pixels or image regions based on a set of features including intensity, texture, and location, using an ensemble of decision trees [10]. As a result of its randomized feature selection and data sampling during training, this algorithm is highly resistant to overfitting and performs well on small datasets and tasks with limited computational demands [11]. The application of AI algorithms has the potential to accelerate the detection of subtle changes in CSF volume and identify potentially dangerous conditions, enabling more timely and effective clinical decision-making [12]. Automating these processes may not only improve diagnostic accuracy but also reduce response times, which can be crucial in urgent cases where timely intervention significantly affects treatment outcomes.

The use of AI for cerebrospinal fluid analysis on head CT represents a new and rapidly developing field of research. A synthesis of the current literature is therefore needed to outline the state of knowledge, summarize recent advances, and identify remaining challenges. In this review, we analyze studies employing AI for the segmentation and volumetric assessment of cerebrospinal fluid in head CT images, with the aim of evaluating whether these methods can support improvements in patient care during the diagnostic process of neurological diseases, as well as assess their potential benefits for radiology and diagnostic imaging professionals through process automation and workflow acceleration.

## 2. Materials and Methods

### 2.1. Search Strategy

In February 2025, a comprehensive search of medical databases was conducted, including MEDLINE via PubMed, Scopus, Web of Science, Embase, and the Cochrane Library.

The search strategy was developed in accordance with the PICO framework [13]. A detailed search strategy and the PICO scheme are provided (Supplement S1).

An initial total of 559 articles was retrieved across all databases. After applying filters, 307 articles remained. These were then imported into the Rayyan tool [14], which automatically identified 98 duplicates, of which 58 were removed.

Subsequently, 249 articles were screened by two independent reviewers. The screening process involved assessing titles, abstracts, and keywords. In total, 232 articles were excluded for the following reasons: wrong publication type (*n* = 18), inappropriate study design (*n* = 199), and paediatric population (*n* = 18).

Ultimately, 14 articles were included in the review (Supplement S2). All reviewer decisions were documented as follows: Reviewer 1—excluded: 224, included: 19, maybe: 6; Reviewer 2—excluded: 229, included: 6, maybe: 19. Discrepancies were resolved by a third independent reviewer. Inter-rater agreement was assessed using Cohen’s Kappa coefficient [15], which yielded a value of 0.56 (agreement rate: 84.36%), interpreted as moderate agreement.

### 2.2. Inclusion and Exclusion Criteria

Inclusion criteria: original, peer-reviewed, full-text articles published in scientific journals within the last 10 years, specifically in the period from 2015 to 2025 with an available abstract, focusing on the assessment of cerebrospinal fluid (CSF) volume and distribution in head CT images using artificial intelligence (AI).

Exclusion criteria: systematic reviews, meta-analyses, conference abstracts, congress proceedings, posters, preprints, letters, studies involving paediatric populations, and any publication types other than original research articles. Studies utilizing imaging modalities other than CT (e.g., MRI); studies older than 10 years; studies not involving AI; studies not focused on head CT; and studies in which CSF was not measured using AI techniques.

### 2.3. Data Extraction

All articles meeting the inclusion criteria underwent data extraction by three independent reviewers. From each study, the following information was collected:

- General characteristics: authors, country of origin, publication year, journal name, impact factor (IF), overall aim of the study, and keywords.
- Dataset information: number of unique patients, mean patient age, and number of CT scans assigned to training, validation, and testing sets.
- Artificial Intelligence (AI): characteristics of the AI method used, software utilized for segmentation, and details of image preprocessing.
- Study type: prospective or retrospective.
- Results: Dice Similarity Coefficient (DSC) and correlation coefficients (r) of volumetric measurements.

### 2.4. Quality Assessment

This systematic review was conducted in accordance with the PRISMA 2020 Statement (Supplement S3) and PRISMA 2020 for Abstracts (Supplement S4) [16]. The quality of the included studies was assessed using the following tools: the JBI Checklist for systematic reviews (Supplement S5) [17], AMSTAR 2 (Supplement S6)—scale for critically appraising systematic reviews of healthcare interventions [18] and the Critical Appraisal Skills Programme (CASP)—diagnostic study checklist (Supplement S7) [19].

### 2.5. Risk of Bias Assessment

We applied the QUADAS-2 framework [20] to systematically evaluate methodological quality and potential bias in each included study. Four core domains were assessed—Patient Selection, Index Test, Reference Standard, and Flow and Timing—alongside three applicability dimensions to ensure alignment with our focus on AI-driven CSF segmentation in head CT. Two reviewers independently completed a tailored QUADAS-2 form, which included signaling questions specific to algorithmic segmentation methods. Disagreements were discussed until consensus was reached, and a third reviewer adjudicated any remaining conflicts. Final judgments (Low, High, or Unclear) for each domain are presented in [Supplement S8], providing a transparent basis for interpreting our review’s conclusions.

## 3. Results

### 3.1. Purpose of Research

All articles included in this systematic review used AI for CSF segmentation and analysis. However, they focused on different aspects and had different objectives. The detailed objective of each study is presented in Table 1.

### 3.2. Sample Size and Mean Age of Study Populations

The number of unique patients varied considerably across the included studies. The smallest cohort, described by Andrei Irimia et al., comprised 35 participants [26], while the largest, reported by Rajat Dhar et al., included 738 subjects [31]. The mean age of patients also differed between studies. The lowest average age was reported by James Booker et al. (55 ± 7 years) [29], whereas the highest was observed in the study by Liang Chen et al. (71 ± 14 years) [30]. One study did not report any information regarding the mean age of its patient population [33].

### 3.3. Type of Study

Nearly all of the studies included in this systematic review were retrospective in nature [21,22,23,24,25,26,27,28,30,31,32,33,34]. Only one study, conducted by James Booker et al., followed a prospective design [29].

### 3.4. Characteristics of the AI Technology

The reviewed studies employed various artificial intelligence models, primarily based on machine learning. The most frequently used approaches included Random Forest algorithms [21,25,28,29] and Convolutional Neural Networks (CNN) [22,23,24,27,31]. The most commonly utilized software for image segmentation was SPM12 [23,24,26] and MIPAV [25,29,31]. Three studies did not report the specific software used for segmentation [21,32,34]. A detailed summary of the types of AI algorithms and segmentation software employed across the included studies is provided in Table 2.

### 3.5. Provided Results

Some authors did not provide detailed results about the accuracy of CSF segmentation by the trained AI models [24,28,31,34]. In contrast, Songsaeng et al. related their findings to the effectiveness of the AI model in diagnosing normal-pressure hydrocephalus [24]. The study by Dhar et al. reported results demonstrating high reproducibility of CSF volume measurements by AI across multiple scans (R = 0.98). Additionally, the AI model showed a significant correlation between increasing CSF volume and patient age (R = 0.73) [28]. In another study by Dhar et al., changes in CSF volume were analyzed as a biomarker for early brain edema development. The model’s performance in measuring CSF volume was also assessed by its correlation with patient age (R = 0.69) [31]. Foroushani et al. evaluated their model primarily based on its sensitivity and accuracy in diagnosing malignant brain edema. The models achieved high sensitivity values (up to 100%) but exhibited low accuracy rates (15–32%) [34].

Most authors assessed the AI’s effectiveness in automatic CSF segmentation by comparing the overlap between AI-generated segmentations and the manual CSF measurements performed by experts, typically quantified using the Dice Similarity Coefficient (DSC) [21,22,25,26,27,29,30,32,33]. The highest DSC values were reported in the studies by Dhar [21], Irimia et al. [26], and Liang et al. [30].

Three studies reported the correlation of volumetric measures between AI-based CSF volume estimates and manual measurements [23,25,32]. All these studies demonstrated high correlation coefficients, with the best result (r = 0.99) observed in the study by Yuan et al. [32]. This indicates a strong agreement between CSF volume measurements obtained via AI models and those derived from manual expert segmentation.

Two studies assessed the consistency and reproducibility of CSF volume measurements using the Intraclass Correlation Coefficient (ICC) [22,26]. Puzio et al. compared the agreement between AI-based automatic CSF volume measurements and manual CSF volume measurements [22]. Irimia et al. compared AI-derived automatic CSF volume measurements from head CT scans with those obtained from MRI scans using the FreeSurfer 6.0 software [23].

Across the included studies, AI-based CSF segmentation showed high performance, with DSC ranging from approximately 0.72 to 0.95, volumetric correlations (r) between 0.91 and 0.99, and ICC between 0.61 and 0.96. A summary of these results is provided in Table 3.

Quality assessment using JBI, AMSTAR 2, and CASP indicated that most studies were methodologically sound but limited by retrospective design, single-center cohorts, and incomplete reporting of validation. According to QUADAS-2, the overall risk of bias was judged as moderate, with the highest concerns in patient selection and reference standards. No quantitative meta-analysis was performed because of substantial heterogeneity in study designs, clinical populations, AI architectures, reference standards, and reported outcomes; results were therefore narratively synthesized.

## 4. Discussion

In this article, we present a systematic review concerning the use of AI for segmentation and volumetric assessment of cerebrospinal fluid (CSF) in CT imaging. Various AI models were used in the analyzed studies, predominantly Random Forest algorithms [21,25,28,29] and CNNs [22,23,24,27,31]. The reviewed studies focused on different clinical indications for CSF volume measurement, with ischemic stroke, brain edema, hydrocephalus, and general assessments of white/gray matter and CSF volumes being the most common.

The results presented in the studies (Table 3) include Dice Similarity Coefficient (DSC), Correlation of Volumetric Measures (Pearson’s correlation coefficient, r), and Intraclass Correlation Coefficient (ICC) [22,26]. DSC measures the degree of spatial overlap between the AI-generated segmentation and a reference segmentation (indicating segmentation accuracy), the correlation coefficient assesses volumetric agreement between methods, and ICC evaluates measurement consistency based on variance analysis. The highest DSC values were reported by Dhar, Liang et al., and Sil C et al., indicating highly precise segmentation [21,27,30]. Lower DSC values observed in studies by Puzio et al., Booker et al., and Chen et al. may reflect greater segmentation challenges, more complex cases, or less effective AI algorithms [22,25,29]. High volumetric correlation confirms strong agreement between AI and reference methods, even when DSC is moderate, as seen in the work of Srikrishna et al. [23]. Yuan et al. reported nearly perfect volumetric agreement (r = 0.99), suggesting excellent model calibration for volume estimation [32].

We used the ICC classification established by the researchers, which in the study conducted by Puzio et al. [22] showed high agreement (>0.9) [35]. Regarding DSC, all reviewed studies achieved values above 0.75, with the majority exceeding 0.8, commonly accepted as an acceptable threshold [36]. These results indicate that AI-based segmentation correlates strongly with the manual gold standard used in individual studies. Given the above 80% overlap of correctly segmented areas, AI technologies have the potential to assist clinicians in CSF segmentation on head CT scans in the future. Such automation could reduce the time required for image analysis and limit repetitive tasks currently performed by medical personnel.

Comparable systematic reviews have explored AI’s role in Adaptive Radiotherapy (ART) for head and neck cancers [37], AI-driven segmentation in Cone-Beam CT (CBCT) imaging of the jaw and mandible [38] and AI-assisted detection of intracerebral hemorrhages in non-contrast CT scans of patients with acute stroke [39]. These studies report DSC values ranging from 0.73 to 0.92, similar to those observed in our review. This suggests that AI applications are advancing not only in CSF analysis and segmentation but also across other areas of medical imaging diagnostics.

Therefore, it can be concluded that AI-based methods could in the future serve as supportive tools for clinicians in identifying pathological conditions by assessing CSF volume changes over time. However, clinical implementation should be deferred until these technologies undergo more rigorous testing and refinement. Although some studies have reported DSC values exceeding 0.9 for segmentation accuracy relative to manual standards, these results were generally obtained on relatively small patient cohorts (e.g., 38 [21], 35 [26], 133 [30] patients). A slightly larger group of patients was included in the study with the highest R-value (0.99) (244 patients) [32]. Additionally, as this review included only peer-reviewed full texts, a potential publication bias may be present.

To achieve reliable outcomes and enable clinical application, prospective, standardized studies in larger patient populations are required. We further recommend standardizing performance metrics across groups, leveraging Big Data to build larger and more diverse datasets, and clearly defining the clinical utility for specific neurological conditions. Finally, clinical adoption will depend on practical integration into routine workflows (PACS/RIS interoperability, predictable turnaround times) and compliance with regulatory pathways for software as a medical device.

## 5. Conclusions

This systematic review of 14 studies highlights CNNs and Random Forest models for automated segmentation and volumetric analysis of CSF on head CT scans. Across the included studies, AI-driven methods consistently achieved high segmentation accuracy (as demonstrated by high DSC) and strong agreement with manual measurements, indicating their potential in neuroradiology.

The current evidence is limited by relevant heterogeneity in datasets and algorithms, retrospective study designs, and relatively small patient cohorts, as well as a lack of prospective clinical validation. To translate these promising findings into routine practice, future research must focus on methodological standardization (including unified segmentation protocols and performance metrics), larger multi-center datasets, and well-designed prospective trials to confirm its utility. Finally, the integration of validated AI tools into everyday radiological workflows could accelerate detection of subtle CSF changes, support clinical decision-making, and enhance overall patient care in neuroimaging.

## Figures and Tables

**Table 1:** Aims of the included studies.

Authors	Aim of the Method
Rajat Dhar [21]	Development of a neural network-based image segmentation algorithm that can automatically measure CSF volume on serial CT scans from stroke patients.
Tomasz Puzio et al. [22]	Assessment on whether CSF distribution evaluated by a specifically developed deep-learning neural network (DLNN) could assist in quantifying mass effect.
Meera Srikrishna et al. [23]	Development of an automatic method for segmenting grey matter (GM), white matter (WM), cerebrospinal fluid (CSF), and intracranial volume (ICV) from head CT images.
Dittapong Songsaeng et al. [24]	Improvement of normal-pressure hydrocephalus diagnosis by comparing the accuracy, sensitivity, specificity, and predictive values of radiological parameters, as evaluated by radiologists and AI methods, utilizing cerebrospinal fluid volumetry.
Yasheng Chen et al. [25]	Development and validation of an automated technique for CSF segmentation via integration of random forest (RF) based machine learning with geodesic active contour (GAC) segmentation.
Andrei Irimia et al. [26]	Evaluation of AI performance in segmenting white matter, gray matter, and cerebrospinal fluid from head CT images.
Sil C. Van De Leemput et al. [27]	Development of a fully convolutional neural network (CNN) for 3D multiclass segmentation in 4D head CT, trained end-to-end using sparse 2D annotations.
Rajat Dhar et al. [28]	Development of a machine learning algorithm capable of segmenting and measuring CSF volume from serial CT scans of stroke patients.
James Booker et al. [29]	Describing the relationship between blood and CSF volumes in different compartments on baseline CT after aSAH, assess whether they independently predict long-term outcomes with AI integration, and explore their interaction with age.
Liang Chen et al. [30]	Development of a novel CNN architecture, called Dense-Res-Inception Net (DRINet), to improve feature extraction and enhance segmentation accuracy in medical images, particularly in cases where differences between categories are subtle in terms of intensity, location, shape, and size.
Rajat Dhar et al. [31]	Assessment on whether early changes in cerebrospinal fluid volume, measured using a neural network-based algorithm, can serve as an early biomarker for cerebral edema and poor clinical outcomes in stroke patients.
Jane Y Yuan et al. [32]	Development of an automated algorithm to extract selective sulcal volume (SSV) and evaluate the age-dependent relationship of reduced SSV on early outcomes after a SAH.
Kevin T. Huang et al. [33]	Development of an algorithm that can automatically detect ventriculomegaly on head CT scans, serving as an indicator of shunt failure in real-life adult hydrocephalus patients. The algorithm aims to achieve this by accurately identifying the lateral and third ventricles.
Hossein Mohammadian Foroushani et al. [34]	Development and evaluation of machine learning models, specifically fully connected and long short-term memory (LSTM) neural networks, to predict which stroke patients will require hemicraniectomy or die due to midline shift. These models use serial clinical and imaging data, including volumetric cerebrospinal fluid (CSF) measurements extracted from baseline and 24-h CT scans.

**Table 2:** Detailed characteristics of the AI and segmentation software.

Authors	Characteristics of the AI Technology	Software Used for Segmentation
Rajat Dhar [21]	Random forest model, which was refined by training a fully CNN (based on the U-Net architecture) to accurately perform the segmentation of CSF	-
Tomasz Puzio et al. [22]	Convolutional neural network with basic U-Net architecture in a 3D version	Exhibeon3 DICOM viewer
Meera Srikrishna et al. [23]	U-Net deep learning model	SPM12
Dittapong Songsaeng et al. [24]	Modified 2D U-Net model	SPM12
Yasheng Chen et al. [25]	Random forest classifiers using Haar-like features combined with geodesic active contour (GAC) refinement via the level-set method	MIPAV
Andrei Irimia et al. [26]	Gaussian Mixture Model (GMM)-based segmentation approach adapted from probabilistic classification methods, incorporating topology-constrained segmentation inspired by Ashburner and Friston’s methods and refined using Bayesian inference principles and a priori tissue probability maps	SPM 12 and MATLAB
Sil C. Van De Leemput et al. [27]	3D CNN architecture, inspired by U-Net	VCAST (volumetric cluster annotation and segmentation tool)
Rajat Dhar et al. [28]	Random forest machine learning	XNAT (eXtensible Neuroimaging Archive Toolkit)
James Booker et al. [29]	Random forest machine learning	MIPAV
Liang Chen et al. [30]	Dense-Res-Inception Net (DRINet)	MRICron
Rajat Dhar et al. [31]	Convolutional neural network (based on the U-Net architecture)	MIPAV
Jane Y Yuan et al. [32]	Deep learning-based approach: a four-layer U-Net	-
Kevin T. Huang et al. [33]	Two-dimensional U-Net	3D Slicer
Hossein Mohammadian Foroushani et al. [34]	A fully connected neural network and a recurrent neural network that employed a Long Short-Term Memory (LSTM) architecture	-

**Table 3:** Results.

Authors	Dice Similarity Coefficient (DSC)	Correlation of Volumetric Measures (r)	Intraclass Correlation Coefficient (ICC)
Dhar [21]	0.95	-	-
Puzio et al. [22]	0.782 (training); 0.765 (validation)	-	0.96
Srikrishna et al. [23]	0.75	0.91	-
Chen et al. [25]	0.751 ± 0.059 (baseline scans); 0.721 ± 0.064 (follow-up scans)	0.92	-
Irimia et al. [26]	0.92 ± 0.007 (study group)/ 0.74 ± 0.066 (control group)	-	0.74 (study group)/ 0.61 (control group)
Sil C. Van De Leemput et al. [27]	0.86 ± 0.04	-	-
Booker et al. [29]	0.7604 ± 0.106	-	-
Liang et al. [30]	0.92	-	-
Yuan et al. [32]	0.82 ± 0.11	0.99	-
Huang et al. [33]	0.809 ± 0.094	-	-

## Data Availability

The original contributions presented in the study are included in the article/Supplementary Materials; further inquiries can be directed to the corresponding authors.

## Supplementary Materials

The following supporting information can be downloaded at: https://www.mdpi.com/article/10.3390/brainsci15111144/s1, Supplement S1. PICO. Supplement S2. PRISMA flow diagram. Supplement S3. PRISMA 2020 checklist. Supplement S4. PRISMA 2020 abstract checklist. Supplement S5. JBI checklist. Supplement S6. AMSTAR2. Supplement S7. CASP checklist. Supplement S8. QUADAS 2—Risk Of Bias Assessment.
