# Supplementary material for: Utilizing Artificial Intelligence for CSF Segmentation and Analysis in Head CT Imaging: A Systematic Review
Source: Brain Sci. 2025 Oct 25;15(11):1144. doi: 10.3390/brainsci15111144 (PMC12650701; doi:10.3390/brainsci15111144)
Supplement: Supplementary file 1 [file brainsci-15-01144-s001.zip › Supplement S1_ PICO.pdf]

**PICO elements**	**Keywords**	**Search terms**	**Search strategy**
**P** (**P**atient or/and **P**opulation)	Adult patients with a head CT scan.	Head CT<br>Brain CT	Head CT<br>OR<br>Brain CT<br>OR<br>Head Computed Tomography<br>OR<br>Brain Computed Tomography<br>OR<br>Head CAT scan<br>OR<br>Brain CAT scan<br>OR<br>Head Computed X-Ray Tomography<br>OR<br>Brain Computed X-Ray Tomography
**I** (**I**ntervention)	Measuring the cerebrospinal fluid volume and distribution on head CT scans.	Cerebrospinal fluid volume<br>Cerebrospinal fluid distribution	Cerebrospinal fluid<br>OR<br>Cerebrospinal fluid volume<br>OR<br>Cerebrospinal fluid distribution
**C** (**C**omparison)	-		
**O** (**O**utcome)	The result of measuring the cerebrospinal fluid volume and distribution on head CT scans using artificial intelligence.	Artificial intelligence	Artificial intelligence<br>OR<br>AI<br>OR<br>Machine Learning<br>OR<br>Deep Learning<br>OR<br>Neural Network<br>OR<br>Computer Neural Network
