# Supplementary material for: Utilizing Artificial Intelligence for CSF Segmentation and Analysis in Head CT Imaging: A Systematic Review
Source: Brain Sci. 2025 Oct 25;15(11):1144. doi: 10.3390/brainsci15111144 (PMC12650701; doi:10.3390/brainsci15111144)
Supplement: Supplementary file 1 [file brainsci-15-01144-s001.zip › Supplement S7_ CASP checklist.pdf]

Critical Appraisal
Skills Programme

CASP Checklist: 10 questions to help you make sense of a **Systematic Review**

How to use this appraisal tool: Three broad issues need to be considered when appraising a systematic review study:

- Are the results of the study valid? (Section A)
- What are the results? (Section B)
- Will the results help locally? (Section C)

The 10 questions on the following pages are designed to help you think about these issues systematically. The first two questions are screening questions and can be answered quickly. If the answer to both is "yes", it is worth proceeding with the remaining questions. There is some degree of overlap between the questions, you are asked to record a "yes", "no" or "can't tell" to most of the questions. A number of italicised prompts are given after each question. These are designed to remind you why the question is important. Record your reasons for your answers in the spaces provided.

About: These checklists were designed to be used as educational pedagogic tools, as part of a workshop setting, therefore we do not suggest a scoring system. The core CASP checklists (randomised controlled trial & systematic review) were based on JAMA 'Users' guides to the medical literature 1994 (adapted from Guyatt GH, Sackett DL, and Cook DJ), and piloted with health care practitioners.

For each new checklist, a group of experts were assembled to develop and pilot the checklist and the workshop format with which it would be used. Over the years overall adjustments have been made to the format, but a recent survey of checklist users reiterated that the basic format continues to be useful and appropriate.

Referencing: we recommend using the Harvard style citation, i.e.: *Critical Appraisal Skills Programme (2018). CASP (insert name of checklist i.e. Systematic Review) Checklist. [online] Available at: URL. Accessed: Date Accessed.*

©CASP this work is licensed under the Creative Commons Attribution – Non-Commercial-Share A like. To view a copy of this license, visit http://creativecommons.org/licenses/by-nc-sa/3.0/ www.casp-uk.net

Paper for appraisal and reference:...

Section A: Are the results of the review valid?

Question		Answer	Hint
1. Did the review address a clearly focused question?	Yes	x	HINT: An issue can be 'focused' In terms of • the population studied • the intervention given • the outcome considered
	Can't Tell		
	No		

Comments:

The review clearly focused on the use of Artificial Intelligence specifically for CSF segmentation and analysis in head CT imaging, highlighting its relevance in neurological diagnostics.

Question		Answer	Hint
2. Did the authors look for the right type of papers?	Yes	x	HINT: 'The best sort of studies' would • address the review's question • have an appropriate study design (usually RCTs for papers evaluating interventions)
	Can't Tell		
	No		

Comments:
Authors searched appropriate databases (MEDLINE, Scopus, Web of Science, Embase, Cochrane Library) for original studies published in the last 10 years, focusing specifically on AI applications for CSF segmentation in head CT imaging, thus making the search appropriate.

Is it worth continuing?

3. Do you think all the important, relevant studies were included?

Yes	x
Can't Tell	
No	

HINT: Look for
- which bibliographic databases were used
- follow up from reference lists
- personal contact with experts
- unpublished as well as published studies
- non-English language studies

Comments:

The authors executed a robust and meticulous search strategy across five major, reputable databases (MEDLINE/PubMed, Scopus, Web of Science, Embase, and Cochrane Library). They started with an extensive initial pool of 559 studies using clearly defined inclusion and exclusion criteria. The dual-reviewer screening, coupled with conflict resolution by a third independent reviewer, further strengthens the reliability and comprehensiveness of the selection process.

4. Did the review's authors do enough to assess quality of the included studies?

Yes	x
Can't Tell	
No	

HINT: The authors need to consider the rigour of the studies they have identified. Lack of rigour may affect the studies' results ("All that glisters is not gold" Merchant of Venice – Act II Scene 7)

Comments:
Quality assessment followed recognized standards such as PRISMA 2020, JBI Checklist, AMSTAR 2, and CASP Diagnostic Study Checklist, indicating a robust and systematic approach to quality appraisal.

5. If the results of the review have been combined, was it reasonable to do so?

Yes	x
Can't Tell	
No	

HINT: Consider whether
- results were similar from study to study
- results of all the included studies are clearly displayed
- results of different studies are similar
- reasons for any variations in results are discussed

Comments:
Combining results based on Dice Similarity Coefficient (DSC), correlation coefficients (r), and Intraclass Correlation Coefficient (ICC) was reasonable, as these metrics consistently measured segmentation accuracy and precision across included studies.

Section B: What are the results?

6. What are the overall results of the review?

HINT: Consider
- If you are clear about the review's 'bottom line' results
- what these are (numerically if appropriate)
- how were the results expressed (NNT, odds ratio etc.)

Comments:
AI demonstrated high accuracy and efficiency in CSF segmentation with promising Dice similarity scores (generally above 0.75), strong correlations in volumetric measurements, and good repeatability. This suggests AI's valuable potential for clinical neurological imaging diagnostics.

7. How precise are the results?

HINT: Look at the confidence intervals, if given

Comments:
Studies reported high Dice coefficients (e.g., up to 0.95), excellent volumetric measurement correlations (up to r=0.99), and very good ICC values (>0.9), reflecting precise and reliable segmentation by AI models.

Section C: Will the results help locally?

8. Can the results be applied to the local population?

Yes	
Can't Tell	x

HINT: Consider whether
- the patients covered by the review could be sufficiently different to your population to cause concern

No [ ] • your local setting is likely to differ much from that of the review

Comments:
Although results are promising, most included studies were retrospective with relatively small patient groups based on different populations. Only one study is based on the Polish population. Prospective validation on larger and more diverse populations is necessary for broader clinical applicability.

9. Were all important outcomes considered?

Yes	x
Can't Tell	
No	

HINT: Consider whether
- there is other information you would like to have seen

Comments:
Important segmentation accuracy outcomes (DSC, correlation of volumetric measures, ICC) were thoroughly considered. However, clinical outcomes, such as the direct impact on patient management and clinical decision-making processes, require further exploration.

10. Are the benefits worth the harms and costs?

Yes	x
Can't Tell	

HINT: Consider
- even if this is not addressed by the review, what do **you** think?

No

Comments:
The review suggests significant potential benefits, including improved diagnostic efficiency and accuracy. Although initial investment and implementation costs could be substantial, the improved diagnostic speed and accuracy potentially outweigh these considerations. However, thorough cost-effectiveness analyses in future research are recommended.
