# Supplementary material for: Utilizing Artificial Intelligence for CSF Segmentation and Analysis in Head CT Imaging: A Systematic Review
Source: Brain Sci. 2025 Oct 25;15(11):1144. doi: 10.3390/brainsci15111144 (PMC12650701; doi:10.3390/brainsci15111144)
Supplement: Supplementary file 1 [file brainsci-15-01144-s001.zip › Supplement S8_ QUADAS 2 - Risk Of Bias Assessment.pdf]

Study	Patient Selection	Index Test	Reference Standard	Flow & Timing	Applicability: Patient Selection	Applicability: Index Test	Applicability: Reference Standard
Booker et al. (2024)	Low	Low	Unclear	Low	Low	Low	Low
Chen et al. (2016)	Low	Low	Low	Low	Low	Low	Low
Chen et al. (2019)	Low	Low	Low	Low	Low	Low	Low
Dhar et al. (2018)	Low	Low	Low	Low	Low	Low	Low
Dhar (2020)	Low	Unclear	Unclear	Unclear	Low	Low	Low
Dhar et al. (2020)	Low	Low	Low	Low	Low	Low	Low
Foroushani et al. (2023)	Low	Low	Low	Low	Low	Low	Low
Huang et al. (2024)	High	Low	Low	High	Low	Low	Low
Irimia et al. (2019)	Low	Low	Low	Low	Low	Low	Low
van de Leemput et al. (2019)	Low	Low	Unclear	Low	Low	Low	Low
Puzio et al. (2022)	Low	Low	Low	Low	Low	Low	Low
Songsaeng et al. (2023)	Unclear	Unclear	Unclear	Unclear	Low	Low	Low
Srikrishna et al. (2022)	Low	Low	Low	Low	Moderate	Low	Moderate
Yuan et al. (2021)	Low	Low	Low	High	Low	Low	Low
